# Clinical profile, patient experience, and management of vitiligo in Italy: Data from the National 2024 Vitiligo Week Awareness Campaign

**DOI:** 10.1016/j.jdin.2026.06.007

**Published:** 2026-06-20

**Authors:** Elisa Zavattaro, Chiara Airoldi, Vanessa Tarantino, Silvia Alberti Violetti, Paolo Amerio, Marco Ardigò, Giuseppe Argenziano, Luca Bianchi, Gabriele Biondi, Alessandra D’Amore, Stefano Dastoli, Elia Esposto, Maria Concetta Fargnoli, Caterina Foti, Giulia Gasparini, Luigi Gargiulo, Giulia Giorgione, Giampiero Girolomoni, Fabrizio Guarneri, Paolo Iacovelli, Cristina Magnoni, Angelo Valerio Marzano, Luca Mastorino, Valentina Mastrandrea, Giuseppe Micali, Maria Letizia Musumeci, Alberico Motolese, Maddalena Napolitano, Lucrezia Pacetti, Andrea Paro Vidolin, Giovanni Pellacani, Maria Antonietta Pilla, Nicola Pimpinelli, Alessandro Pileri, Bianca Maria Piraccini, Ilaria Proietti, Eugenio Provenzano, Pietro Quaglino, Marco Romanelli, Franco Rongioletti, Pietro Rubegni, Giovanni Sarracco, Massimiliano Scalvenzi, Sara Scrivani, Oriana Simonetti, Luca Stingeni, Iris Zalaudek, Leonardo Zichichi, Francesca Zottarelli, Paola Savoia

**Affiliations:** a Department of Health Science, University of Eastern Piedmont, Novara, Italy; b SCDU Dermatologia, Azienda Ospedaliero-Universitaria Maggiore della Carità di Novara, Novara, Italy; c Department of Pathophysiology and Transplantation, Università degli Studi di Milano, Milano, Italy; d Università G. D’Annunzio, Chieti-Pescara; e Humanitas University, Pieve Emanuele, Milano; f University L. Vanvitelli, Napoli; g Dermatology Unit, Tor Vergata University Hospital, Roma; h Clinica Dermatologica, AOU di Sassari; i Department of Dermatology, Catholic University of the Sacred Heart, Roma; j Department of Health Sciences, University Magna Graecia of Catanzaro; k San Gallicano Dermatological Institute IRCCS, Roma; l Section of Dermatology, Department of Precision and Regenerative Medicine and Jonian Area, University "Aldo Moro" of Bari; m Section of Dermatology, Department of Health Sciences (DISSAL), University of Genova & Dermatology Unit, Ospedale Policlinico San Martino IRCCS, Genova; n AOUI Ospedale Borgo Trento, Verona; o AOU Policlinico G. Martino, Messina; p Department of Dermatology, University of Modena and Reggio Emilia, Modena & Azienda Unità Sanitaria Locale, Modena, Italy; q Ospedale Dermatologico San Lazzaro, Torino; r Azienda Ospedaliero Universitaria Policlinico di Bari, Bari, Italy; s Dermatology Clinic, University of Catania, Catania, Italy; t Arcispedale Santa Maria Nuova, Reggio Emilia; u AOU Federico II, Napoli; v UO Dermatologia, Arcispedale Sant’Anna di Cona, Ferrara; w Ospedale Israelitico Sede Isola Tiberina, Roma; x Policlinico Umberto I, Roma; y Istituto Dermopatico dell’Immacolata, Roma; z SC Dermatologia, Dip. Scienze della Salute Università degli Studi di Firenze & Azienda USL Toscana Centro, Firenze, Italy; aa Policlinico S. Orsola, Bologna; bb Unit of Dermatology, Department of Medical-Surgical Science and Biotechnologies, Sapienza University of Rome, A. Fiorini Hospital, Latina, Italy; cc Presidio Ospedaliero Mariano Santo, Cosenza; dd AOU Ospedale Santa Chiara, Pisa; ee IRCCS Ospedale San Raffaele, Milano; ff Ospedale Le Scotte, Siena; gg AO S. Pio presidio G. Rummo, Benevento; hh Ospedale Maggiore, Parma; ii Clinica Dermatologica, Università Politecnica delle Marche, Ancona, Italy; jj Dermatology Section, Department of Medicine and Surgery, University of Perugia; kk Department of Dermatology and Venereology, University of Trieste, Trieste, Italy; ll Ospedale Sant'Antonio Abate, Erice

**Keywords:** epidemiology, patient awareness, patient survey, psychosocial impact, quality of life, vitiligo

*To the Editor:* Vitiligo is a chronic skin disorder characterized by progressive melanocyte loss, frequently associated with comorbidities, particularly thyroid disease.^1^ Although neither life-threatening nor contagious, its visible manifestations often lead to psychological distress, social stigma, and impaired life quality.^2^ Global prevalence is estimated at 0.5% to 2%, with considerable variation across regions and ethnic groups. However, updated epidemiological data in Italy remain scarce.^3^^,^^4^

The Vitiligo Week campaign, organized by the Italian Society of Dermatology (SIDeMaST) in November 2024, engaged 37 centers nationwide to raise awareness and collect clinical and patient-reported data. A geographical map of participating centers is shown in Supplementary Fig 1 (available via Mendeley at https://www.mendeley.com/reference-manager/library/all-references/3f12037e-3952-3618-94a1-d9d03133e7a9). Participants completed a self-administered questionnaire followed by a dermatological evaluation. Associations between psychological burden and covariates were evaluated using univariable and multivariable logistic regression. Analyses were conducted in SAS 9.4 and R, with statistical significance set at *P* < .05.

A total of 637 participants were diagnosed with vitiligo (Supplementary Fig 2, available via Mendeley at https://www.mendeley.com/reference-manager/library/all-references/3f12037e-3952-3618-94a1-d9d03133e7a9). Demographic and clinical characteristics are summarized in Supplementary Table I (available via Mendeley at https://www.mendeley.com/reference-manager/library/all-references/3f12037e-3952-3618-94a1-d9d03133e7a9). The cohort was predominantly female and Italian-born, with a mean age of 50.8 years; a family history was reported by about one-third. Most patients had nonsegmental vitiligo (87.8%) and limited disease extent (<10% body surface area in 51.3%). The face/neck (87.6%) and hands (79.9%) were the most frequently involved sites. Previous treatment was reported by 79.1% and consisted mainly of phototherapy and topical corticosteroids, although only 40.9% perceived a benefit. Mean age at onset was 31.6 years, with an average diagnostic delay of 2.1 years; dermatologists established 91.4% of diagnoses (Table I).

**Table I:** Timing, source, and psychosocial impact of vitiligo diagnosis among participants∗

Patients with previous vitiligo diagnosis (*N* = 605)			
Age at first onset, y (*N* = 560)		Negative impact on life and relationship (*N* = 602)	
Mean (SD)	31.58 (16.05)	Yes	396 (65.78%)
Min-max	0-80	No	206 (34.22%)
Age at diagnosis, y (*N* = 507)		Impact on context (*N* = 396)	
Mean (SD)	33.71 (15.81)	Family	70 (17.68%)
Min-max	1-80	Partner	79 (19.95%)
Difference between first onset and diagnosis, y (*N* = 442)		Friends	195 (49.24%)
Median (Q1; Q3)	1 (0; 2)	Work environment/school	248 (62.63%)
Min-max	0-46	Personal aspects	32 (8.08%)
Diagnoses performed by (*N =* 605)		Social interactions	18 (4.55%)
Dermatologist	553 (91.40%)	Societal impact	20 (5.05%)
General practitioner	61 (10.08%)	Summer-related impact	13 (3.28%)
Other	11 (1.82%)	Other	39 (9.84%)
		Strength of the impact (*N* = 392)∗	
		Not at all	9 (2.27%)
		A little	89 (22.47%)
		Somewhat	184 (46.46%)
		Very	71 (17.93%)
		Extremely	39 (9.85%)

*max*, maximum; *min*, minimum; *Q*, quartile.

∗ Only positive responses (yes) are presented.

Psychological impact was reported by 65.8% of patients, affecting primarily work/school (62.6%) and friendships (49.2%). Although severe distress was infrequent (9.9%), the high prevalence of psychosocial burden highlights the broader impact of vitiligo beyond visible symptoms. In multivariable models, predictors of greater psychological burden included female sex, older age, residence in Southern Italy/Islands, darker phototype (IV/V), and comorbidities (Table II). These findings underscore the need for holistic management approaches addressing both physical and emotional well-being and suggest that psychosocial support may be particularly relevant across diverse populations, extending beyond the Italian context.

**Table II:** Vitiligo impact and its association with demographic and clinical covariates∗

	No impact (*n* = 206)	Negative impact (*n =* 396)	OR (95% CI) univariable	*P* value	OR (95% CI) multivariable	*P* value
	*N* (%)	*N* (%)				
Sex						
Female vs male	107 (51.94%)	264 (66.67%)	1.85 (1.31; 2.61)	.0005	1.58 (1.04; 2.41)	.0315
Age at diagnosis, y						
≤20	45 (25.14%)	74 (22.56%)	1 (ref)	.6198		
20-40	78 (43.58%)	138 (42.07%)	1.08 (0.68; 1.71)			
>40	56 (31.28%)	116 (35.37%)	1.26 (0.77; 2.05)			
Age at visit, y						
≤20	8 (4.19%)	5 (1.37%)	1 (ref)	.0008	1 (ref)	.0114
20-40	53 (27.75%)	61 (16.67%)	1.84 (0.57; 5.97)		1.46 (0.41; 5.23)	
>40	130 (68.06%)	300 (81.97%)	3.69 (1.18; 11.49)		2.77 (0.81; 9.46)	
Geographical origin						
North	80 (38.83%)	149 (37.63%)	1 (ref)	.0358	1 (ref)	.0660
Center	79 (38.35%)	120 (30.30%)	0.82 (0.55; 1.21)		0.71 (0.44; 1.14)	
South/ Islands	47 (22.82%)	127 (32.07%)	1.45 (0.94; 2.23)		1.29 (0.79; 2.11)	
Sites involved						
Visible vs nonvisible areas	195 (94.66%)	384 (96.97%)	1.81 (0.78; 4.17)	.1662		
Phototype						
I/II	70 (39.33%)	166 (49.85%)	1 (ref)	.0236	1 (ref)	.0307
III	103 (57.87%)	151 (43.35%)	0.62 (0.43; 0.90)		0.65 (0.43; 0.98)	
IV/V	5 (2.81%)	16 (4.80%)	1.35 (0.48; 3.82)		2.03 (0.63; 6.58)	
Comorbidities						
Yes vs no	76 (36.89%)	206 (52.02%)	1.85 (0.31; 2.62)	.0004	1.63 (1.08; 2.48)	.0214

∗ *N* (%) indicates the number of subjects in each category, reported separately for no and negative impact.

This nationwide study is the largest contemporary investigation of vitiligo in Italy, combining clinical data with patient-reported outcomes. The high prevalence of active disease and visible lesions highlights the critical need for early intervention, regular monitoring, and personalized management. Diagnostic delays and modest treatment efficacy reveal ongoing gaps in care. The psychosocial burden is substantial, particularly among women and residents of Southern regions, reflecting both the visibility of lesions and sociocultural factors. Overall, these findings underscore the urgent importance of integrating mental health support into routine vitiligo care.^5^

Limitations include missing data for selected variables and voluntary participation, with 45% of available screening slots remaining unfilled. This may have introduced selection bias, as individuals with more severe disease or greater health awareness may have been more likely to participate. Nonetheless, the study offers critical insights into the clinical and emotional burden of vitiligo in Italy and underscores the importance of increasing public awareness, ensuring timely specialist access, and implementing comprehensive strategies that address both medical and psychological dimensions of care.

## Conflicts of interest

None disclosed.
